# Surgery as a life transition: A qualitative study of patient experiences of person-centred care

**DOI:** 10.1177/17504589261451117

**Published:** 2026-06-23

**Authors:** José Miguel Seguro, Francisco Matos, Inês Martins Esteves, Márcia Pestana-Santos

**Affiliations:** 1 Health Sciences Research Unit: Nursing (UICISA: E), Coimbra, Portugal; 2 Nursing School of University of Coimbra (ESEUC), Coimbra, Portugal; 3 Sanfil Medicina, Coimbra, Portugal; 4 University of Coimbra, Coimbra, Portugal; 5 Unidade Local Saúde de Coimbra, Coimbra, Portugal; 6 School of Medicine and Biomedical Sciences, University of Porto, Porto, Portugal; 7 RISE-Health, Faculty of Medicine of University of Porto, Porto, Portugal

**Keywords:** Qualitative research, Person-centred care, Patient experience, Perioperative care, Transitions theory

## Abstract

**Introduction:**

Surgery represents a profound life event rather than merely a clinical procedure, yet comprehensive accounts of the entire perioperative journey remain scarce, particularly in Southern European contexts.

**Objectives:**

This qualitative study explored how adult patients experience perioperative care in a Portuguese hospital, interpreting findings through Meleis’ Transitions Theory.

**Methods:**

Eighteen elective surgery patients participated in semi-structured interviews postoperatively. Thematic analysis revealed that participants experienced surgery as interconnected transitions in which emotional, informational and relational care dimensions proved as vital as technical competence.

**Results:**

Eight themes emerged across the surgical journey: preparation, information processing, safety protocols, recovery room awakening, home transition, staff connections, waiting and uncertainty, and postsurgical reflection. Empathetic communication, consistent nursing presence and personalised support fostered security and control, whereas rushed explanations and inadequate follow-up amplified vulnerability.

**Conclusion:**

Conceptualising perioperative care as transitions reveals critical nursing intervention points. Structured communication, emotional support, carer engagement and tailored discharge planning are essential components of quality surgical nursing, transforming uncertainty into readiness and facilitating smoother recovery.

## Introduction

### Background

For patients, surgery represents far more than a technical procedure confined to the operating theatre. It constitutes a deeply personal journey, characterised by anticipation, vulnerability and the challenge of navigating unfamiliar clinical environments while coping with physical discomfort and emotional uncertainty. Over the past two decades, surgical care has progressively moved beyond a narrow biomedical focus towards models that acknowledge the psychological, social and relational dimensions of patient experience (Bolton & Gillett 2019, Wade & Halligan 2017, Yu et al 2023).

Person-centred care, broadly understood as care that respects and responds to individual needs, values and preferences, has become widely recognised as a hallmark of high-quality perioperative nursing (Arakelian et al 2017, McCance & McCormack 2025). This approach integrates clinical expertise with empathetic communication, shared decision-making and continuity across different phases of care. Research has linked person-centred approaches to improved patient satisfaction, enhanced quality of life and better recovery outcomes (Calabro et al 2018, Coulter & Oldham 2016, Engle et al 2021, Renghea et al 2022).

Despite widespread endorsement at the policy level, however, everyday perioperative practice frequently falls short of these ideals. Patients continue to report rushed consent discussions, inadequate preparation for what lies ahead and limited emotional support when it is most needed. Such gaps can intensify anxiety, undermine confidence and potentially compromise recovery (Gobbo et al 2020, Wu et al 2024). Addressing these shortcomings requires more than technical proficiency; it demands relational competence, thoughtful communication and genuine partnership with patients and their families throughout the surgical pathway.

### Theoretical framework

Meleis’ Transitions Theory offers a particularly useful lens through which to conceptualise the surgical journey (Meleis 2010, Meleis et al 2000). Rather than viewing this journey as a series of disconnected clinical episodes, the theory invites us to understand it as a sequence of interlinked transitions, each carrying distinct vulnerabilities and opportunities for adaptation.

Contextualising this theory within person-centred surgical care highlights its practical utility. The core components of the theory map directly onto the known priorities of perioperative nursing. For instance, a patient’s ‘Transition Conditions’, such as personal knowledge and emotional readiness, are directly shaped by the quality of preoperative information and empathetic communication. ‘Patterns of Response’ illustrate the patient’s journey from a state of vulnerability and anxiety towards a state of mastery and confidence, a trajectory heavily influenced by ‘Nursing Therapeutics’. These therapeutic interventions include providing clear explanations during safety checks, offering a reassuring presence in the recovery room and delivering personalised discharge planning. By framing surgical care through this lens (Meleis 2010, Meleis et al 2000), the study identifies specific, actionable nursing interventions that can strengthen person-centred practice and facilitate smoother transitions.

The success with which patients navigate these phases depends upon conditions that include their knowledge, available social support and emotional readiness. Patient responses may range from anxiety and confusion to a growing sense of mastery. Nurses and, in the United Kingdom, Operating Department Practitioners (ODPs) are uniquely positioned to shape these transitions through interventions such as clear communication, coping support and involvement of family members in care (Dahlberg et al 2024, Gustavell et al 2025, Nilsson 2019).

### Study rationale and aim

While international studies have examined various aspects of perioperative care, holistic accounts that follow patients across the entire surgical pathway, from preoperative preparation through to home discharge, remain uncommon in the qualitative evidence base, which is often fragmented by clinical setting or phase of care (van Grootel et al 2025). Evidence from Southern Europe, particularly Portugal, is especially sparse. This gap is notable given the unique cultural and systemic context of the Portuguese health care landscape. In Portugal, there is a strong cultural expectation of family involvement in caregiving, yet the hospital system, particularly in the private sector, is increasingly influenced by efficiency-driven, short-stay surgical models. Understanding how patients navigate the tension between familial support and clinical efficiency during transitions is essential for developing culturally sensitive, person-centred care.

Therefore, the aim of this study was to explore how adult patients experience perioperative care in a Portuguese private hospital and to interpret how informational, emotional and relational factors influence their adaptation across the surgical continuum, guided by Meleis’ Transitions Theory.

## Methods

### Design

A qualitative exploratory-descriptive design was selected, as it enables researchers to capture participants’ perspectives on complex experiences without imposing a rigid theoretical framework from the outset (Hunter et al 2019). This approach afforded the flexibility to describe perioperative experiences in rich detail while drawing upon Meleis’ Transitions Theory as an interpretive lens during analysis. Meleis’ Transitions Theory provided the conceptual foundation, proposing that health and illness are characterised by transitions, with outcomes shaped by three interrelated elements: transition conditions (e.g. knowledge and support), patterns of response (such as vulnerability and mastery) and nursing interventions (including presence and guidance) (Meleis 2010, Meleis et al 2000).

### Research question and objectives

The primary research question guiding this study was:

**Research Question:** How do adult patients experience care transitions throughout the perioperative journey in a Portuguese private hospital?

This was supported by two specific objectives: (1) to explore the informational, emotional and relational factors that shape patient adaptation during the surgical process and (2) to interpret patient experiences and care transitions through the theoretical lens of Meleis’ Transitions Theory.

### Sample and setting

The study was conducted in the surgical wards of a private hospital in Portugal between September and October 2024. The unit comprised 37 beds distributed across single, double and quadruple rooms. Purposive sampling was employed to ensure variation in age, sex, surgical specialty, American Society of Anesthesiologists (ASA) physical status classification and length of postoperative hospital stay. Recruitment was guided by the principle of data saturation, a point where no new information or themes emerge from the data, a standard approach for ensuring adequacy in qualitative inquiry (Vasileiou et al 2018).

### Inclusion and exclusion criteria

Eligible participants were adults aged 18 years or older scheduled for elective inpatient surgery under general or regional anaesthesia, fluent in Portuguese and able to provide informed consent. Individuals with cognitive impairment, those undergoing outpatient surgery and those whose surgery addressed complications from a previous procedure were excluded. Of the 20 patients approached, 18 agreed to participate.

### Ethical considerations

Ethical approval was granted by the relevant institutional ethics committee (reference number P1039_05_2024). Written and oral informed consent was obtained from all participants. Participation was entirely voluntary and had no impact on clinical care. Anonymity and confidentiality were protected through numerical identifiers and the removal of all personal details. No member of the research team with access to the data had direct clinical responsibilities for the participants. Data were stored securely on password-protected, encrypted devices, and participants were informed that they could amend or withdraw their data at any stage before analysis. A referral protocol for psychological distress was in place, although its activation was not required.

### Data collection

Participants were identified from daily surgical ward lists and approached in person by the interviewer, who was a nurse with postgraduate training in qualitative research, no clinical responsibilities in the unit and no prior relationship with participants. Individual semi-structured interviews were conducted on the first or second postoperative day in a private room. The interview guide (Supplementary File 1) was informed by existing literature and reviewed by an expert panel. Interviews lasted between 11 and 32 min, with an average duration of 19 min. All interviews were audio-recorded with consent and transcribed verbatim in Portuguese. Transcripts were checked for accuracy by a second researcher. English translations were undertaken by bilingual researchers and reviewed by a native English speaker. Recruitment continued until data saturation was reached, which became apparent after 15 interviews, with three further interviews conducted to confirm saturation.

### Data analysis

Analysis followed Braun and Clarke’s six phases of thematic analysis (Braun & Clarke 2012, Byrne 2022). Two researchers independently coded transcripts, compared their coding, resolved discrepancies through discussion and developed a shared coding framework. Themes were refined iteratively through team discussions. All coding decisions, theme development processes and analytic reflections were documented in an audit trail. No software was used. Rigour was addressed using Lincoln and Guba’s criteria of credibility, dependability, confirmability and transferability (Nowell et al 2017) through researcher triangulation, reflexive journaling and detailed contextual reporting. This study is reported in accordance with the COnsolidated criteria for REporting Qualitative Research (COREQ) 32-item checklist (Tong et al 2007) (Supplementary File 2).

## Findings

### Participant characteristics

Eighteen participants took part in the study, with a median age of 55 years (range = 48–62 years). The group included nine women and nine men, representing a range of surgical specialties, ASA classifications and lengths of postoperative stay. Table 1 provides a detailed overview of participant characteristics.

**Table 1:** Demographic and clinical characteristics of participants

Characteristics	*n* (%) or *median* [IQR]
Sex	
Female	9 (50.0)
Male	9 (50.0)
Age (years)	55 [48-62]
Type of surgery	
General surgery	4 (22.2)
Gynaecology surgery	1 (5.6)
Urology surgery	1 (5.6)
Orthopaedics surgery	9 (50.0)
Plastic surgery	2 (11.1)
Vascular surgery	1 (5.6)
Type of anaesthesia	
General anaesthesia	10 (55.6)
Regional anaesthesia	8 (44.4)
Past surgeries	1.72 [1-2]
Moment of admission	
Surgery day	13 (72.2)
Day before surgery	5 (27.8)
ASA classification	
ASA I	2 (11.1)
ASA II	10 (55.6)
ASA III	6 (33.3)
Length of postoperative hospital stay (days)	2.8 [2-4]

### Overview of themes

The analysis generated one overarching theme, ‘Patients’ perioperative experience as a life transition’, supported by eight interrelated themes spanning the preoperative, intraoperative, postoperative and discharge phases (Figure 1). Participants consistently acknowledged the technical competence of staff but emphasised that informational, emotional and relational factors shaped their journey as significantly as clinical outcomes. Each theme illuminates moments of both vulnerability and adaptation, resonating with Meleis’ Transitions Theory. Table 2 provides a comprehensive overview of themes, subthemes, illustrative quotes, practice implications and theoretical linkages.

**Figure 1:**
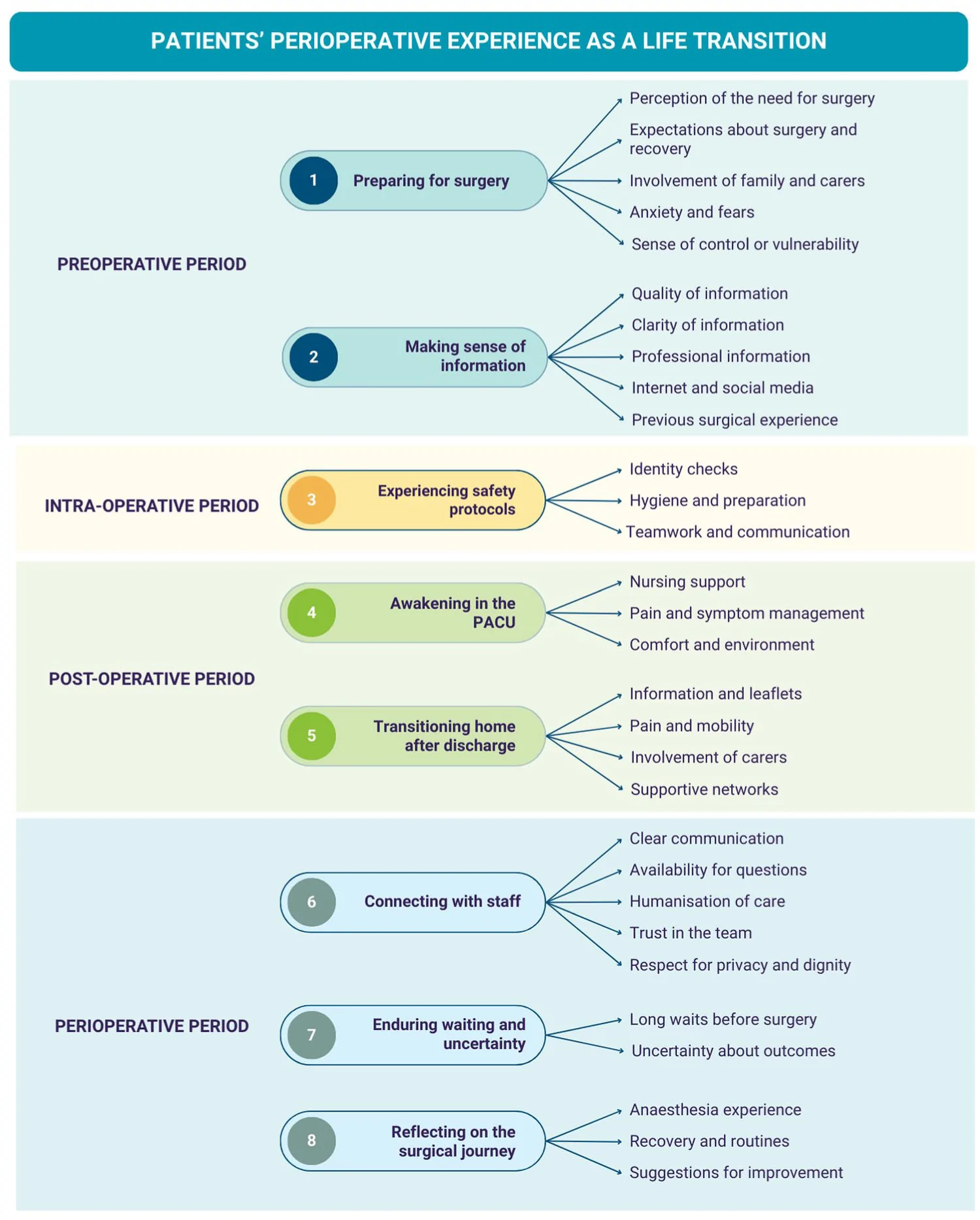
Conceptual map of themes and subthemes illustrating patients’ perioperative experience *Source*: Authors 2026

**Table 2:** Overview of themes, subthemes, exemplar quotes and their implications for practice and theory.

Theme	Subtheme	Exemplar quote(s)	Practice implications (what to do)	Transitions theory linkage
Preparing for surgery	Perception of the need for surgery	‘*The doctors said it had to be done, so I just trusted them*’ (Interview #11)	Ensure rationale for surgery is clearly explained; encourage questions; support informed decision-making.	*- Nature of transition* (health-illness) - *Transition conditions* (trust, comprehension) *- Patterns of response* (engagement, confidence)
	Expectations about surgery and recovery	‘*They* [healthcare team] *explained everything: what could go well, what might not . . . it helped me feel in control*’ (Interview #18)	Provide balanced, realistic, personalised expectations; discuss potential complications and recovery timelines.	*- Transition conditions* (knowledge, preparedness) *- Patterns of response* (sense of control)
	Involvement of family and carers	‘*My children live abroad . . . I had to go through this on my own*’ (Interview #17)	Involve carers in consultations; arrange alternative support for patients without family nearby.	*- Transition conditions* (social support) *- Patterns of response* (connectedness, isolation)
	Anxiety and fears	‘*I was afraid of what I’d feel when I woke up . . . would I be in pain? Would everything go as planned? You never really know*’ (Interview #11)	Screen for preoperative anxiety; teach coping strategies and offer emotional support.	*- Transition conditions* (emotional well-being) *- Patterns of response* (anxiety, readiness)
	Sense of control or vulnerability	‘*They explained each step–that made me feel safe*’ (Interview #06)	Give step-by-step updates; reduce waiting without information; maintain patient agency.	*- Transition conditions* (clarity, involvement) *- Patterns of response* (empowerment, vulnerability)
Making sense of information	Quality of information	‘*They* [surgical team] *didn’t try to make it sound perfect, but they also didn’t scare me*’ (Interview #18)	Deliver honest, balanced information; avoid minimising risks or overemphasising benefits.	*- Transition conditions* (communication quality) *- Patterns of response* (trust)
	Clarity of information	‘*At the preoperative appointments, they* [the healthcare team] *explained everything to me: what to expect before, during, and after the surgery*’ (Interview #15)	Use plain language; avoid jargon; reinforce with written or visual aids.	*- Transition conditions* (knowledge) *- Patterns of response* (preparedness vs. confusion)
	Professional information	‘*They explained every step to me, and that really reassured me*’ (Interview #06)	Allocate time for Q&A; ensure consistent messaging across staff.	*- Transition conditions* (staff accessibility) *- Patterns of response* (reassurance)
	Internet and social media	‘*I did a lot of research before deciding. It helped me feel like I was in control*’ (Interview #08)	Provide patients with curated, trustworthy online resources; caution against misinformation.	*- Transition conditions* (resource availability) *- Patterns of response* (empowerment vs. confusion)
	Previous surgical experience	‘*I’ve been through surgery before, so I had a good idea of what to expect*’ (Interview #14)	Explore past surgical experiences; clarify differences in current procedure; correct assumptions.	*- Nature of transition* (cumulative experience) *- Patterns of response* (confidence vs. false assumptions)
Experiencing safety protocols	Identity checks	‘*They asked me several times about my name and which side they were operating . . . I realised it was just a safety measure*’ (Interview #18)	Explain the purpose of safety checks to patients; use them as opportunities for reassurance.	*- Transition conditions* (process clarity) *- Patterns of response* (security)
	Hygiene and preparation	‘*They* [ward nurses] *gave me a special soap . . . and explained that it helps prevent infections*’ (Interview #12)	Always explain infection-prevention measures and their importance; invite questions.	*- Transition conditions* (education, engagement) *- Patterns of response* (shared responsibility)
	Teamwork and communication	‘*Everyone had a job, and they worked like clockwork. It made me feel like I was in good hands*’ (Interview #09)	Maintain visible teamwork; give proactive updates during delays.	*- Transition conditions* (team coordination, information flow) *- Patterns of response* (trust, reassurance vs. anxiety)
Awakening in the PACU	Nursing support	‘*I don’t remember much, but I do remember a nurse’s voice asking how I was feeling . . . it made me feel like I wasn’t alone*’ (Interview #07)	Offer verbal reassurance immediately upon patient awakening; maintain presence.	*- Patterns of response* (emotional safety v vulnerability) *- Transition conditions* (timely presence)
	Pain and symptom management	‘*As soon as I mentioned it, they gave me something, and the pain faded*’ (Interview #11)	Respond promptly to reported pain or symptoms; check proactively symptoms.	*- Patterns of response* (comfort, trust vs. distress) *- Transition conditions* (symptom management)
	Comfort and environment	‘*It was hard to relax with all the beeping and movement around me, but I knew I was being monitored*’ (Interview #13)	Offer orientation cues; explain alarms and surroundings to reduce disorientation; manage comfort.	*- Transition conditions* (environmental familiarity) *- Patterns of response* (reassurance vs. disorientation)
Transitioning home after discharge	Information and leaflets	‘*They gave me a booklet with all the instructions . . . at home, I could go back and check it when I had doubts*’ (Interview #09)	Provide written and verbal discharge instructions; allow time for patient and carer questions.	*- Transition conditions* (information continuity) *- Patterns of response* (self-management vs. uncertainty)
	Pain and mobility	‘*Even getting out of bed was a challenge*’ (Interview #12) ‘*The pain was manageable because they explained exactly what to take and when*’ (Interview #05)	Set realistic expectations; provide clear medication schedules; discuss expectations for pain control; address mobility issues.	*- Transition conditions* (skill acquisition, expectation management) *- Patterns of response* (confidence vs. struggle)
	Involvement of carers	‘*My wife accompanied me and received some guidance, but. . . they could involve her more*’ (Interview #02) ‘*Most of the time I was on my own*’ (Interview #17)	Actively include carers in discharge planning; provide tailored guidance; identify isolated patients; activate community support.	*- Transition conditions* (support system) *- Patterns of response* (connectedness vs. isolation)
	Supportive networks	‘*I live alone . . . I didn’t really know where to turn for extra help*’ (Interview #17)	Arrange follow-up calls, community nursing or volunteer services postdischarge.	*- Transition conditions* (resource availability) *- Patterns of response* (support vs. abandonment)
Connecting with staff	Clear communication	‘*They explained things in a way I could understand–no complicated medical terms*’ (Interview #04)	Use accessible language; check understanding; avoid overload.	*- Transition conditions* (communication quality) *- Patterns of response* (confidence, control)
	Availability for questions	‘*I asked a lot of questions, and they never seemed annoyed*’ (Interview #07) ‘*I. . . kept some of my questions to myself*’ (Interview #11)	Encourage questions; signal approachability despite time pressures.	*- Patterns of response* (engagement vs. hesitations) *- Transition conditions* (supportive relationships) - *Nursing interventions* (supportive presence)
	Humanisation of care	‘*One of the nurses held my hand . . . that little gesture calmed me more than anything else*’ (Interview #08)	Incorporate small, compassionate gestures into routine care.	*- Patterns of response* (emotional comfort) *- Transition conditions* (nurse-patient connection) - *Nursing interventions* (comforting presence)
	Trust in the team	‘*The way they worked together gave me confidence*’ (Interview #12) ‘*Everyone had a job, and they worked like clockwork*’ (Interview #09) ‘*I trusted them completely*’ (Interview #05)	Make teamwork visible; clarify roles; communicate with confidence.	*- Transition conditions* (team functioning) *- Patterns of response* (trust, reassurance)
	Respect for privacy and dignity	‘*They always asked for my permission before doing anything*’ (Interview #03) ‘*Shared rooms sometimes compromised confidentiality*’ (Interview #14)	Seek consent before interventions; mitigate privacy risks in shared spaces.	*- Transition conditions* (autonomy) *- Patterns of response* (empowerment vs. compromised dignity)
Enduring waiting and uncertainty	Long waits before surgery	‘*I was admitted . . . but my surgery only happened late at night. . . that waiting just made me more nervous*’ (Interview #08) ‘*It would have been good to get more information*’ (Interview #11)	Provide proactive updates; explain delays; set timeframes for the next information.	*- Transition conditions* (information flow) *- Patterns of response* (anxiety, uncertainty)
	Uncertainty about outcomes	‘*I just hope this surgery helps . . . what if the pain comes back? What if it doesn’t work as expected?* ’ (Interview #06)	Discuss possible outcomes and contingencies preoperatively; normalise uncertainty.	*- Transition conditions* (realistic preparation) *- Patterns of response* (hope vs. worry)
Reflecting on the surgical journey	Anaesthesia experience	‘*I remember lying there . . . and suddenly I was out. . . when I woke up, it was all over*’ (Interview #05) ‘*I wasn’t expecting that, but I guess they did what was best for me*’ (Interview #18)	Provide clear preoperative anaesthesia briefings; address potential changes in plan.	*- Transition conditions* (anticipatory guidance) *- Patterns of response* (reassurance, acceptance)
	Recovery and routines	‘*I thought I’d bounce back quickly, but the reality was different. The first days were though*’ (Interview #09) ‘*Gradual return to routines was celebrated*’ (Interview #7)	Set realistic recovery timelines; validate early struggles; support graded return to activity.	*- Transition conditions* (expectation management) *- Patterns of response* (adaptation)
	Suggestions for improvement	‘*If they had kept us updated while we were waiting, it would have helped a lot*’ (Interview #18) ‘*It would be great if there was some kind of home care service*’ (Interview #06)	Implement real-time updates; personalise discharge planning; explore short-term home care support.	*- Patterns of response* (engagement, satisfaction) *- Transition conditions* (resources, communication)

Q&A: questions and answers.

### Theme 1: Preparing for surgery

Preparation shaped both understanding of the procedure and emotional readiness. Clear, personalised explanations helped reduce anxiety. As one participant noted:

They explained everything . . . it helped me feel in control. (P18)

Family presence emerged as a critical source of reassurance. Those without support nearby felt the absence keenly:

My children live abroad . . . I had to go through this on my own. (P17)

From a transitions’ perspective, preoperative preparation represents a crucial transition condition, where adequate information and social support enhance patients’ readiness to navigate the surgical experience.

### Theme 2: Making sense of information

Access to information directly shaped trust and feelings of preparedness. Patients valued balanced accounts that neither minimised risks nor exaggerated benefits:

They didn’t try to make it sound perfect, but they also didn’t scare me. (P18)

Dense, jargon-filled explanations were overwhelming, whereas professional advice carried particular weight. This theme reflects the transition condition of knowledge, with clear communication serving as a nursing intervention that facilitates adaptation.

### Theme 3: Experiencing safety protocols

When staff explained their purpose, repeated checks became a source of reassurance:

It made me feel safer. (P18)

Without explanation, patients simply complied passively. Teamwork and coordination inspired confidence:

Everyone had a job, and they worked like clockwork. (P9)

Visible teamwork functioned as a relational intervention, reinforcing patients’ sense of being in capable hands during the intraoperative transition.

### Theme 4: Awakening in the recovery room

Even a brief nursing presence carried profound meaning during the vulnerable moment of emergence from anaesthesia:

I do remember a nurse’s voice asking how I was feeling . . . it made me feel like I wasn’t alone. (P7)

Timely pain relief was central to building trust. The environment was often described as functional but impersonal, contributing to confusion. This finding illustrates Meleis’ patterns of response, where relational presence facilitates movement from vulnerability towards mastery.

### Theme 5: Transitioning home after discharge

Written materials supported recall and provided a reference point. However, rushed conversations left patients with unanswered questions:

When I got home, I realised I had questions. (P14)

Pain and mobility limitations were often underestimated, and carer involvement was inconsistent. Discharge emerged as a particularly vulnerable transition in which inadequate preparation compromised patients’ ability to adapt to the post-hospital environment.

### Theme 6: Connecting with staff

Clear, jargon-free explanations helped patients feel in control, while compassionate gestures carried disproportionate weight:

One of the nurses held my hand . . . that calmed me more than anything else. (P8)

Respect for dignity was emphasised repeatedly, although shared rooms sometimes compromised confidentiality. Such moments exemplify nursing interventions that address emotional and relational dimensions of care.

### Theme 7: Enduring waiting and uncertainty

Operational delays without updates were deeply stressful:

My surgery only happened late at night. I had no idea when it would be my turn. (P8)

While patients understood that emergencies take priority, the absence of communication was distressing. From a transitions’ perspective, unexplained waiting represents a modifiable condition that undermines readiness and heightens vulnerability.

### Theme 8: Reflecting on the surgical journey

Recovery was slower and more challenging than many had anticipated. Yet gradual return to normal routines was celebrated:

Each day I could do a little more . . . that felt like an achievement. (P7)

Suggestions for improvement included real-time updates during delays, more personalised discharge plans and short-term home care support, all pointing towards nursing interventions that could strengthen future patients’ capacity to navigate surgical transitions.

## Discussion

This study explored how adult patients experience perioperative care in a private hospital in Portugal, revealing that emotional, informational and relational dimensions mattered as much as technical quality in shaping the surgical journey. Across eight interrelated themes, patients consistently valued empathy, clear communication and continuity of nursing presence, hallmarks of person-centred care (Coulter & Oldham 2016, Havana et al 2023, Sundqvist et al 2018).

Viewing these findings through Meleis’ Transitions Theory, the surgical pathway emerges as a sequence of interconnected transitions, each representing both vulnerability and opportunity for adaptation (Meleis 2010, Meleis et al 2000). This aligns with international evidence showing that effective transitions require attention to both clinical and psychosocial dimensions (Nilsson 2019, Nilsson et al 2020).

Preoperative preparation emerged as a critical stage where personalised, realistic information reduced uncertainty and fostered readiness, echoing recent work by Dahlberg et al (2024). Clear, jargon-free communication repeatedly emerged as a key factor in patients feeling in control, reinforcing evidence that person-centred communication must be accurate, paced and reinforced across stages of care (Danaher et al 2023, Krist et al 2017). Visible safety procedures reassured participants only when their purpose was explained, aligning with Lindqvist Leonardsen et al (2024).

Recovery room narratives emphasised the disproportionate reassurance provided by even brief nursing presence, supporting earlier work by Nilsson (2019) and illustrating Meleis’ patterns of response, where mastery is facilitated by relational care. This finding underscores the importance of nursing presence during moments of heightened vulnerability, suggesting that even brief, intentional contact can meaningfully influence patients’ emotional states and their capacity to navigate the immediate postoperative transition.

Discharge emerged as a particularly vulnerable transition, especially for those without strong social support networks. Generic discharge instructions left some patients unprepared for the realities of pain and limited mobility at home, echoing a recent meta-synthesis by van Grootel et al (2025). Carer involvement was inconsistent despite evidence that engaging family members improves self-care confidence and reduces readmission rates (Engle et al 2021, van Grootel et al 2025). Proactive inclusion of carers and structured follow-up align closely with person-centred principles (Gonçalves Bradley et al 2022, Handiyani et al 2024).

Operational challenges, particularly prolonged waiting without updates, intensified anxiety and eroded trust. From a transitions’ perspective, these represent modifiable conditions that undermine readiness, pointing to opportunities for structured communication protocols (Gustavell et al 2025). The distress caused by unexplained waiting suggests that even when delays are unavoidable, transparent communication can mitigate their psychological impact.

Across all phases of care, three elements of Transitions Theory were consistently evident. First, transition conditions, such as readiness enhanced by personalised information or compromised by rushed communication. Second, patterns of response, where mastery was supported by relational presence and distress heightened by uncertainty. Third, nursing interventions, including clear explanations, emotional reassurance, coping support and carer involvement. Together, these findings highlight opportunities for targeted nursing interventions that could strengthen perioperative transitions and improve recovery outcomes (Meleis 2010).

### Strengths and limitations

This study has several strengths. Purposive sampling captured diverse surgical specialties, enhancing the transferability of findings. Adherence to COREQ standards supports transparency and rigour. Bilingual translation with validation by a native English speaker helped ensure linguistic and conceptual equivalence. Triangulation of analysis by multiple researchers strengthened interpretive depth.

Several limitations should be acknowledged. The single-site setting in a private hospital may not reflect experiences in public health care systems or other cultural contexts. The sample included only patients undergoing elective surgery; experiences may differ for emergency procedures. Interviews were conducted within 2 days postoperatively, meaning accounts may have been influenced by residual effects of anaesthesia, analgesia or fatigue (Dahlberg et al 2024). Although the interviewer had no clinical responsibilities in the unit, his institutional affiliation may have influenced participant responses, although reflexivity practices were employed to minimise this.

### Implications for practice

This study points to practical strategies for embedding person-centred care into routine surgical pathways. Structured communication protocols, proactive coping support and tailored discharge planning should be standardised rather than left to individual initiative. Family and carer involvement must be consistently integrated into perioperative processes, not treated as optional extras. Hospitals should provide evidence-based educational resources that patients can access before and after surgery, complemented by follow-up contacts or community nursing visits where appropriate.

Specifically, the following practice recommendations emerge from these findings:

- Preoperative preparation should include personalised, realistic information about the surgical journey, delivered in accessible language and reinforced through written materials.
- Intraoperative communication should explain the purpose of safety protocols, transforming passive compliance into active reassurance.
- Recovery room care should prioritise nursing presence, however brief, to support patients during the vulnerable emergence from anaesthesia.
- Discharge planning should begin preoperatively, involve carers where available and include structured follow-up to address questions and challenges that arise after home return.
- Waiting periods should be accompanied by regular updates, even when no new information is available, to mitigate anxiety and preserve trust.

### Implications for research

Future research should evaluate the effectiveness of nurse-led interventions such as anxiety-reduction programmes and personalised discharge planning, particularly in resource-constrained systems like the Portuguese National Health Service. Comparative studies across public and private settings, as well as across different cultural contexts, would illuminate how organisational and cultural factors shape perioperative transitions. Longitudinal research following patients beyond the immediate postoperative period would provide insight into longer-term adaptation and recovery trajectories.

## Conclusions

For patients undergoing elective inpatient surgery, perioperative experiences were shaped as much by emotional, informational and relational factors as by clinical care itself. Clear communication, empathetic presence and continuity of support during transitions fostered feelings of safety, control and readiness for recovery. Integrating person-centred principles into surgical pathways requires more than technical excellence; it demands proactive emotional support, realistic expectation setting, meaningful carer engagement and tailored discharge planning. Applying Meleis’ Transitions Theory provides a framework for identifying key vulnerabilities across the perioperative continuum and designing targeted nursing interventions to address them.

## Supplemental Material

sj-docx-1-ppj-10.1177_17504589261451117 – Supplemental material for Surgery as a life transition: A qualitative study of patient experiences of person-centred care Supplemental material, sj-docx-1-ppj-10.1177_17504589261451117 for Surgery as a life transition: A qualitative study of patient experiences of person-centred care by José Miguel Seguro, Francisco Matos, Inês Martins Esteves and Márcia Pestana-Santos in Journal of Perioperative Practice
