# Supplementary material for: Surgery as a life transition: A qualitative study of patient experiences of person-centred care
Source: J Perioper Pract. 2026 Jun 23;36(11):676–87. doi: 10.1177/17504589261451117 (PMC13683895; doi:10.1177/17504589261451117)
Supplement: Supplementary file 1 [file sj-docx-1-ppj-10.1177_17504589261451117.docx]

**Supplementary File 1**

**Semi-Structured Interview Guide**

This guide was used to explore participants’ experiences of perioperative care, from preoperative preparation to discharge. Questions were informed by existing literature on perioperative experiences and patient-centred care, and refined through expert review in perioperative nursing, clinical leadership, and qualitative methodologies.

Each question was asked in a flexible order depending on the flow of the conversation. Prompts and follow-up questions were used as appropriate to encourage elaboration and clarify responses.

**Preoperative Phase**

1. What is your assessment of the information you received before your surgery?
2. What do you think about the information regarding the care you should take before, during, and after surgery?
3. What were you told about potential risks and complications?
4. What other information would you have liked to have had beforehand about your surgery?
5. Was there anything you would have liked to have known beforehand that was not mentioned? Please describe.
6. Overall, how would you describe your preparation for surgery? What could have been done to improve your experience?

**Intraoperative Phase**

7. How would you describe the care you received before, during, and after surgery?
8. Can you describe how you perceived respect for your self-determination and privacy during the process (from the moment you knew you were going to have surgery until you were discharged)?
9. What do you think of the support you received from the interprofessional team (nurses, physicians/surgeons, other professionals)?
10. How would you describe the professionals’ concern for your comfort and well-being?
11. Did you notice if the surgical team used checklists or safety protocols during the process?
12. Were there any situations in which you felt safety or communication could have been optimised? If so, what was it?

**Postoperative Phase and Discharge**

13. How prepared do you feel for discharge from the hospital?
14. What concerns or challenges do you anticipate facing in the post-discharge period?
15. What kind of help do you think you will need? Is it already guaranteed?
16. How have your family and/or caregiver(s) been included in the process? Do you feel they have received enough information to support you in the postoperative period?
17. What concerns or challenges do you anticipate facing in the post-discharge period?

Note: This full interview guide is referenced in the *Data Collection* subsection of the Methods. It is provided here to enhance transparency, reproducibility, and adherence to COREQ reporting standards.
